# Proteomic analysis of plasma to identify novel biomarkers for intra-amniotic infection and/or inflammation in preterm premature rupture of membranes

**DOI:** 10.1038/s41598-023-32884-y

**Published:** 2023-04-06

**Authors:** Ji Hyun Back, So Yeon Kim, Man Bock Gu, Hyeon Ji Kim, Kyong-No Lee, Ji Eun Lee, Kyo Hoon Park

**Affiliations:** 1 Department of Biotechnology, College of Life Sciences and Biotechnology, Korea University, Seoul, 02841 Korea; 2 Biomedical Research Division, Chemical and Biological Integrative Research Center, Korea Institute of Science and Technology, Seoul, 02792 Korea; 3 Department of Obstetrics and Gynecology, University of Ulsan College of Medicine, Asan Medical Center, Seoul, Korea; 4 Department of Obstetrics and Gynecology, Seoul National University College of Medicine, Seoul National University Bundang Hospital, 82, Gumi-Ro 173 Beon-Gil, Bundang-Gu, Seongnam, 463-707 Korea

**Keywords:** Biomarkers, Molecular medicine

## Abstract

To identify potential plasma biomarkers associated with microbial invasion of the amniotic cavity (MIAC) and/or intraamniotic inflammation (IAI) in women with preterm premature rupture of membranes (PPROM). This retrospective cohort study included 182 singleton pregnant women with PPROM (23–33 weeks) who underwent amniocentesis. Plasma samples; all subjects were chosen from these participants and were analyzed using label-free liquid chromatography-tandem mass spectrometry for proteome profiling using a nested case–control study design (cases with MIAC/IAI *vs*. non-MIAC/IAI controls [*n* = 9 each]). Three identified target molecules for MIAC/IAI were further verified by ELISA in the study cohort (*n* = 182). Shotgun proteomic analysis revealed 17 differentially expressed proteins (*P* < 0.05) in the plasma of MIAC/IAI cases. In particular, the levels of FCGR3A and haptoglobin, but not LRP1, were found to be increased in the plasma of patients with MIAC, IAI, and both MIAC/IAI compared with those without these conditions. Moreover, these differences remained significant after adjusting for gestational age at sampling. The area under the curves of plasma FCGR3A and haptoglobin ranged within 0.59–0.65 with respect to each of the three outcome measures. Plasma FCGR3A and haptoglobin were identified as potential independent biomarkers for less-invasively detecting MIAC/IAI in women with PPROM.

## Introduction

Preterm birth affects approximately 10% of all pregnancies and remains the leading cause of neonatal mortality and morbidity worldwide^1^. In particular, preterm premature rupture of membranes (PPROM), which occurs in 3–4% of all deliveries, is an important antecedent to approximately 30% of preterm births^1–3^. PPROM is often complicated by the subclinical presence of microorganisms in the amniotic fluid (AF) (*i.e.*, microbial invasion of the amniotic cavity [MIAC]) and/or intraamniotic inflammation (IAI), with a frequency of approximately 50%^4–6^. A solid body of evidence suggests that the presence of MIAC/IAI is associated with additional risks of adverse pregnancies (*i.e.*, delivery latency) and neonatal short- and long-term outcomes, which may be caused by fetal inflammation and injury to the immature organs^7–10^. Importantly, recent studies have shown that intravenous clarithromycin therapy may reduce the intensity of the intraamniotic inflammatory response in patients with PPROM with either MIAC or sterile IAI^6,11^. Thus, accurate and early identification of women at high risk for MIAC/IAI allows targeted use of novel therapeutics that can substantially reduce the incidence of complications in women with PPROM and their children, as well as improve the use of resources, such as transfer to a tertiary center, and use of corticosteroids and magnesium for neuroprotection.

AF analysis via amniocentesis for various interleukins (ILs) and matrix metalloproteinases remains the gold standard method for identifying MIAC/IAI complicated by PPROM^2,4,8,12,13^. However, this approach is clinically challenging owing to the need of invasive procedures and technical difficulty in some cases of PPROM with severe oligohydramnios secondary to ruptured membranes. In this context, a maternal blood sample, which can be obtained via a less invasive, easy-to-use, and inexpensive method, could be a preferable alternative to AF. Indeed, several studies have shown a significant elevation of various inflammatory proteins that occur concurrently in the AF and maternal blood compartments in the setting of MIAC/IAI and spontaneous preterm delivery (SPTD)^12,14,15^. However, to date, few studies have explored the predictive potential of these blood protein mediators for MIAC/IAI complicated by PPROM, particularly using high-throughput proteomic methodologies.

Mass spectrometry-based shotgun proteomics coupled with multidimensional chromatography separation has recently emerged as a high-throughput technique for characterizing proteomes with low abundance in complex biological samples (plasma and serum)^16^. This approach has shown promising results in the discovery of new protein markers for diseases with complex phenotypes, such as various forms of infection/inflammation^17–21^. The purpose of this study was (i) to identify potential plasma biomarkers associated with MIAC/IAI in women with PPROM using label-free shotgun proteomic analysis and (ii) to determine the top-ranked protein pathways activated under these conditions.

## Materials and methods

### Ethical approval

The study was approved by the local ethics committee of the Seoul National University Bundang Hospital, Seongnamsi, Korea (project number B-1105/128-102). All experiments were performed in accordance with the relevant guidelines and regulations of the ethics committee of the hospital. All participants provided written informed consent to collect and use biological samples and clinical information prior to the amniocentesis procedure.

### Study population and research design

This retrospective study enrolled 182 singleton pregnant women admitted to the Department of Obstetrics and Gynecology at the Seoul National University Bundang Hospital, Seongnamsi, Korea, with a diagnosis of PPROM at 23 + 0 to 33 + 6 weeks of gestation between June 2004 and April 2019. The inclusion criteria were the following: (i) performance of transabdominal amniocentesis to assess possible subclinical intraamniotic infection and inflammation; (ii) delivery of a live fetus; and (iii) availability of a maternal plasma sample collected at the time of the amniocentesis. Participants were excluded if they had (i) active labor (defined as cervical dilation ≥ 4 cm in the presence of uterine contraction by sterile speculum examination), (ii) multiple pregnancies, (iii) a fetus with major congenital anomalies, and (iv) clinical chorioamnionitis at the time of admission. Gestational age was determined using the last menstrual period and ultrasound estimates based on first or second (≤ 20 weeks) trimester fetal biometry. PPROM was defined as clinically confirmed amniorrhexis occurring prior to labor onset and at < 37 weeks of gestation. This condition was diagnosed visually by sterile speculum examination to confirm the pooling of AF in the vagina (or AF leakage through the cervix**)** and a positive nitrazine test (and/or a positive AmniSure ROM test [Qiagen, Hilden, Germany]).

**Figure 1:**
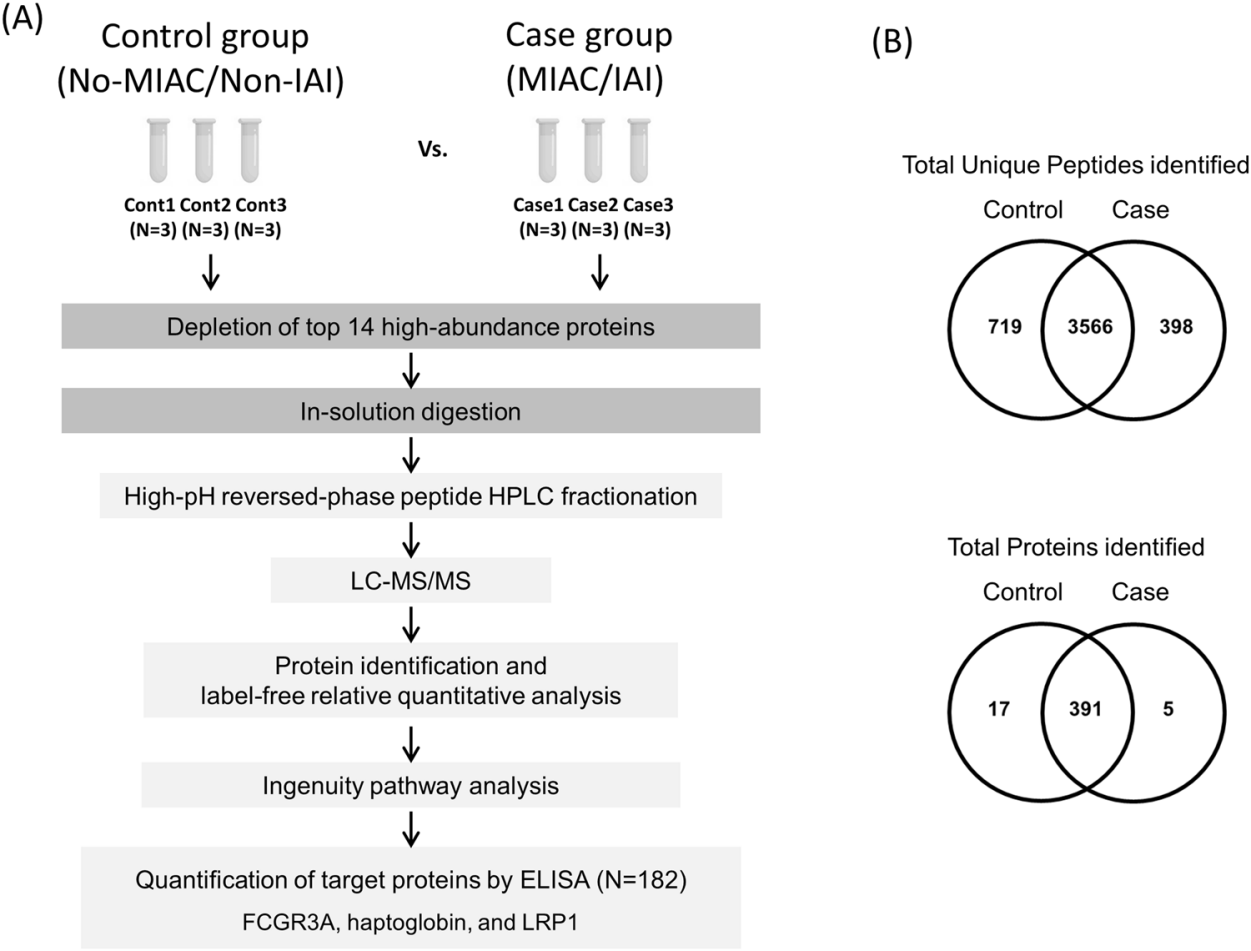
(**A**) Schematic workflow for the discovery (label-free LC–MS/MS) and verification (immunoassay) experiments. Three sets of pooled plasma samples from control (non-MIAC/IAI) and case (MIAC/IAI) groups were subjected to immunoaffinity depletion to remove the 14 most abundant proteins followed by tryptic digestion. Peptides were fractionated with high-pH reversed phase chromatography, after which were subjected to LC–MS/MS followed by label-free quantitative analysis based on peak intensities. Differentially expressed proteins were further investigated by IPA and the DEPs of interest were validated by ELISA. (**B**) Venn diagrams showing the distribution of common and uniquely peptides (above) and proteins (below) identified in the LC–MS/MS analyses. *MIAC* microbial invasion of the amniotic cavity, *IAI* intra-amniotic inflammation, *LC* liquid chromatography *MS/MS* tandem mass spectrometry, *IPA* Ingenuity pathway analysis, *DEP* differentially expressed proteins, *ELISA* enzyme-linked immunosorbent assaym, *FCGR3A* low affinity immunoglobulin gamma Fc region receptor III-A, *LRP1* prolow-density lipoprotein receptor-related protein 1.

For the discovery phase of the study (Fig. 1A), a nested case–control approach was performed, comprising nine patients with MIAC (who also had IAI [case subjects]) and nine gestational age-matched patients without MIAC and IAI (control subjects). The case patients were randomly selected among the 52 patients with MIAC/IAI within the total study cohort of patients with PPROM using a random sequence generator. Each control patient was matched to the case patient in terms of gestational age at sampling, parity, years of admission, and maternal age.

### Diagnosis of MIAC and IAI using AF samples

Ultrasound-guided transabdominal amniocentesis was performed under aseptic conditions at the time of admission. AF samples were sent to the hospital laboratory for culturing aerobic and anaerobic bacteria, genital mycoplasma (*Ureaplasma urealyticum* and *Mycoplasma hominis*), and fungi, as well as for assessing the white blood cell count (WBC), as previously described^22^. The remaining AF was centrifuged for 10 min at 1500 × *g*, and the supernatant was divided into aliquots and stored at − 70 °C until further analysis. The managing physicians had access to the results of the AF analysis (AF culture results and WBC counts, but not IL-6 quantification). MIAC was defined as the presence of a positive AF culture for bacteria, genital mycoplasma (*Ureaplasma* spp*. or M. hominis),* and/or fungi*.*

AF IL-6 levels were assayed using the enzyme-linked immunosorbent assay (ELISA) human IL-6 DuoSet Kit (R&D Systems, Minneapolis, MN, USA) to define subclinical IAI. A detailed description of the measurements of IL-6 concentrations in AF is provided in Supplementary Methods. IAI was diagnosed when the AF IL-6 concentration was ≥ 2.6 ng/mL, as previously described^23–25^.

### Collection and storage of plasma samples

On admission to a hospital, at the time of amniocentesis, prior to the administration of medications, maternal venous blood samples were collected into ethylenediaminetetraacetic acid tubes after measuring the C-reactive protein (CRP) concentration and WBC counts in patients diagnosed with PPROM, as part of the hospital protocol. Plasma was separated from the blood samples by centrifugation at 1500 × *g* at 4 °C for 10 min and stored in multiple aliquots at − 70 °C until further use.

### Management of PPROM and clinical definitions of various factors

Management of PPROM has been previously described in detail^26,27^. Briefly, prophylactic antibiotics (macrolides plus ampicillin) were administered to all patients with PPROM. Tocolytic therapy (magnesium sulfate, ritodrine, or atosiban) and a course of corticosteroid treatment were administered to women with PPROM at 23–34 weeks of gestation at the discretion of the clinician. In patients with PPROM at < 34 weeks of gestation who proved to have positive AF cultures, the decision for delivery was not made only based on the positive AF cultures results; delivery was considered if the woman was diagnosed or suspected of chorioamnionitis or whose fetus was diagnosed or suspected of being in jeopardy. Delivery was considered for all women with PPROM ≥ 34 weeks. Acute histologic chorioamnionitis (HCA) was diagnosed based on the presence of acute inflammation (defined by neutrophil infiltration) in any placental tissue (chorionic plate, umbilical cord, or fetal membranes [chorion-decidua and amnion]) in accordance with previously published criteria^28,29^. Clinical chorioamnionitis was diagnosed using the criteria proposed by Gibbs et al.^30^; more detailed criteria are provided in Supplementary Methods.

### Proteomic analysis (discovery phase)

Protein concentration was determined using a bicinchoninic acid protein assay kit (Thermo Fisher Scientific, Bremen, Germany) in 18 exploratory cohort samples (Fig. 1A). In each case (*n* = 9) and control (*n* = 9) groups, three sets of plasma samples were pooled in equal amounts per sample, thus creating three sets of pooled plasma samples for the case and control groups. These pooled plasma samples were subjected to immunoaffinity depletion to remove 14 high-abundance proteins, tryptic digestion, and high-pH reversed-phase peptide fractionation. The fractionated peptide samples were then analyzed in triplicate by liquid chromatography-tandem mass spectrometry (LC–MS/MS) using an Eksigent MDLC system (Eksigent Technologies, Dublin, CA, USA) interfaced to an LTQ XL-Orbitrap mass spectrometer (Thermo Fisher Scientific, Waltham, MA, USA).

For protein identification, a protein database search was performed against the SwissProt human database (20,329 entries; release date: July 2020) using MaxQuant (version 1.6.7.0)^31,32^. The false discovery rate for peptides and proteins was set at 1%, and all proteins were identified using two or more unique peptides. For quantification purposes, the MaxLFQ algorithm was employed for label-free quantification (LFQ) of the proteins. Proteins with fold change of LFQ intensities > 1.3 or < 0.77 and *P* < 0.05 were considered as differentially expressed proteins (DEPs). Perseus software (1.6.14.0; https://maxquant.net/perseus/) was used for the statistical analysis of the label-free quantitative datasets^33,34^. Detailed descriptions of the discovery proteomics experiments are provided in Supplementary Methods.

### Ingenuity pathway analysis (IPA)

The proteomic dataset, which included UniProt accession numbers of the DEPs and their corresponding log_2_|Fold Change| of LFQ intensities, was submitted to IPA (data version 65,367,011; QIAGEN, Redwood City, CA) for functional analysis to identify the canonical pathways, diseases, and biological functions involved. The uploaded DEPs were mapped to the corresponding gene objects in the Ingenuity Pathways Knowledge Base as a reference set^35,36^. A right-tailed Fisher’s exact test was used to determine the likelihood of the mapped genes in each pathway (network) to be found together due to random chance.

### ELISA (validation phase)

Three selected candidate DEPs were validated in the study cohort, comprising 182 individual samples. The concentrations of haptoglobin (DuoSet ELISA, R&D Systems, Minneapolis, MN, USA), low-affinity immunoglobulin gamma Fc region receptor III-A (FCGR3A), and prolow-density lipoprotein receptor-related protein 1 (LRP1) (MyBioSource, San Diego, CA, USA) were assayed using commercial ELISA kits, according to the manufacturer’s instructions. Aliquots of the frozen plasma were thawed at room temperature (25 °C) for up to 1–2 h and vortexed thoroughly prior to analysis. The plasma dilutions used and the working range for each ESISA kit are described in detail in Supplementary Methods. The intra- and inter-assay coefficients of variation (CVs) were of < 10% for all analyzed proteins, except for the inter-assay CVs of FCGR3A (11.2%) and haptoglobin (11.4%). The above-mentioned three target molecules were selected for the validation study because: i) they revealed a high differential expression (*i.e.*, fold change) or statistical significance; ii) little or no information was available regarding their expression change in the plasma in relation to MIAC/IAI; iii) they could have potential clinical relevance in the plasma with respect to inflammation/infection, considering their biological functions; and iv) ELISA kits for these proteins were readily available. Insulin-like growth factor II (DuoSet ELISA, R&D Systems, Minneapolis, MN, USA) and properdin (complement factor P; MyBioSourceSan Diego, CA, USA) were also assessed in the plasma using spike-and-recovery and linearity-of-dilution testing, but the results revealed poor assay performance; hence, these molecules were not further investigated.

### Statistical analysis

The clinical data and plasma levels of the proteins were compared using Fisher’s exact test or the *χ*^2^-test for categorical data, Student’s *t*-test for normally distributed continuous data, and the Mann–Whitney *U*-test for non-normally distributed continuous data. Multivariate logistic regression was performed to estimate independent associations between plasma levels of each protein and the outcome measures after adjusting for gestational age at sampling and parity, which had a *P*-value < 0.1 in univariate analyses. Additionally, to determine the best blood-based multi-marker panels for MIAC, IAI, and microbial-associated IAI, multivariate analyses with a forward selection were performed on the two newly identified plasma biomarkers (FCGR3A, haptoglobin) along with conventional inflammatory marker in the blood (serum CRP), which were selected based on *P* < 0.1 in univariate analysis. The optimal cutoff and diagnostic value of each candidate protein were assessed through the analysis of their receiver operating characteristic (ROC) and area under the curve (AUCs). Thereafter, pairwise comparisons of the AUCs of each investigated plasma biomarker, serum CRP (as standard of inflammation biomarker), and multi-marker panel were performed using the method proposed by DeLong et al.^37^. The optimal cutoff value was determined using the maximal Youden's index (sum of sensitivity + specificity − 1). Potential interactions between three proteins profiled (FCGR3A, haptoglobin, and LRP1) were assessed using type-III tests. Spearman rank correlation was conducted to assess the linear correlations of each protein level. All probability values were two-sided and *P* < 0.05 were considered significant. Statistical analyses were performed using SPSS version 25.0 (IBM Inc., Armonk, NY, USA).

## Results

### Characteristics of the discovery cohort

The demographic and clinical characteristics of the exploratory cohort used for shotgun proteomics are shown in Table S1. Owing to the matched selection criteria, the cases and controls had similar gestational age at sampling, use of medications, maternal age, and parity. Nine patients with MIAC complicated by PPROM were positive for *U. urealyticum* (*n* = 9), *M. hominis* (*n* = 6), and *Peptostreptococcus* spp. (*n* = 1) as determined by AF culture, among whom 77.7% (7/9) of patients in the MIAC group had polymicrobial invasion.

### Exploratory proteomic analysis

**Figure 2:**
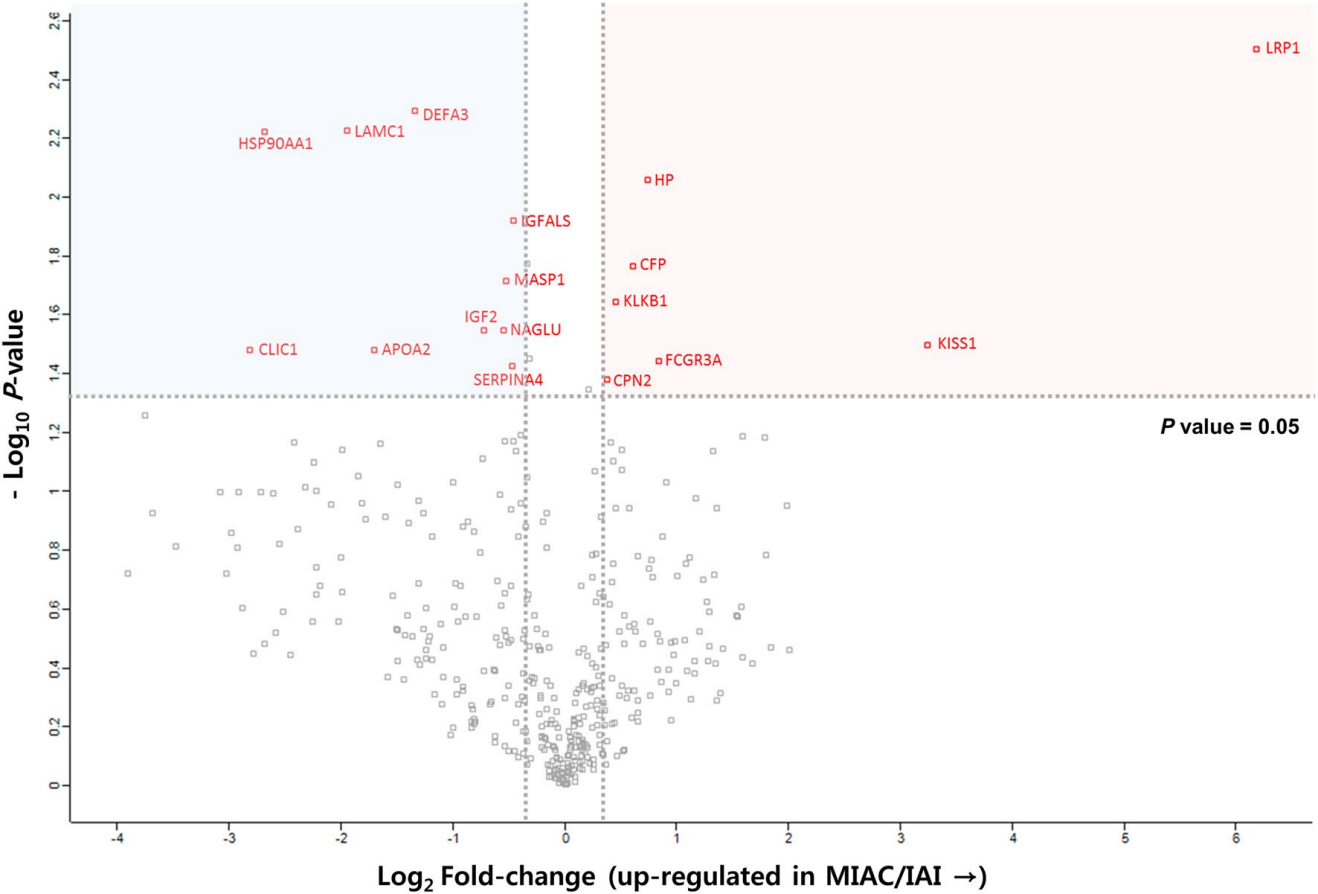
Label-free quantification proteomic data of DEPs in MIAC/IAI case *vs*. non-MIAC/IAI control. Representative protein identifiers in red indicate statistically significant DEPs with *P* < 0.05 and fold change of LFQ intensities > 1.3 or < 0.77. *DEP* differentially expressed proteins, *MIAC* microbial invasion of the amniotic cavity, *IAI* intra-amniotic inflammation, *LFQ* label-free quantification.

Overall, LC–MS/MS analyses of three plasma biological replicates revealed 4285 and 3964 unique peptides in the case and control groups, respectively, whereas 3566 peptides were identified in both groups (Fig. 1B). A total of 408 proteins were identified in the plasma of the control group and 396 in the case group, whereas 391 proteins were present in both groups (Fig. 1B and Table S2). Among these shared proteins, 7 (41.2%) were significantly upregulated and 10 (58.8%) were downregulated in MIAC/IAI cases as compared with cases without these conditions (Fig. 2 and Table S3). Hierarchical clustering analysis of these 17 DEPs further confirmed that their expression pattern in the MIAC/IAI cases was significantly different from that of the No-MIAC/Non-IAI controls (Fig. S1). Analysis of these 17 DEPs using IPA revealed five canonical pathways (Table S4): ‘acute phase response signaling (APR),’ ‘growth hormone signgling,’ ‘iron homeostasis signaling pathway,’ peroxisome proliferator-activated receptors (PPAR)/retinoid X receptor (RXR) activation,’ and ‘hypoxia-inducible factor 1 (HIF-1) signaling.’ IPA also identified ‘inflammatory response,’ ‘cancer,’ ‘organismal injury and abnormalities,’ and ‘metabolic disease’ as the top diseases and disorders associated with the presence of MIAC and IAI complicated by PPROM.

### Verification of proteomic data in the total cohort

In the total study cohort (*n* = 182), the overall rates of MIAC and IAI were 34.6% (63/182) and 42.8% (78/182), respectively, and 28.5% (52/182) of women had both MIAC and IAI (microbial-associated IAI). MIAC and IAI alone were present in 6.0% (11/182) and 14.2% (26/182) of women, respectively, whereas 51% (93/182) of women exhibited neither MIAC nor IAI. Genital mycoplasmas (*U. urealyticum* [*n* = 49] and/or *M. hominis* [*n* = 33]) were the most common microbes found in the AF. Other microorganisms isolated from AF samples included *Streptococcus agalactiae* (*n* = 3), *Peptostreptococcus spp.* (*n* = 3), *Streptococcus viridans* (*n* = 2), *Lactobacillus spp.* (*n* = 2), *Streptococcus mitis* (*n* = 1), *Haemophilus influenzae* (*n* = 1), *Escherichia coli* (*n* = 1), gram-negative rods (*n* = 1), *Candida glabrata* (*n* = 1), and gram-positive cocci (*n* = 1). Polymicrobial findings were present in 55.5% (35/63) of the patients with MIAC.

To verify the proteomic data, the levels of three candidate DEPs, FCGR3A, haptoglobin, and LRP1, were determined in MIAC/IAI cases and then compared with those of controls without these conditions. The median plasma levels of FCGR3A and haptoglobin were significantly higher in women with MIAC, IAI, and microbial-associated IAI than in women without these conditions (*P* < 0.05, Table 1). However, univariate analysis showed that women with MIAC, IAI, and microbial-associated IAI had significantly lower gestational age at sampling and gestational age at delivery than those without these conditions (Table 2). Moreover, women with IAI and microbial-associated IAI were more or tended to be more parous than those without these conditions (Table 2). Additional multivariate analysis further demonstrated that high plasma levels of haptoglobin were significantly associated with MIAC, IAI, and microbial-associated IAI after adjusting for gestational age at sampling and parity (Table 3). Similar associations were observed between FCGR3A and MIAC and microbial-associated IAI**,** whereas no association of FCGR3A with IAI was observed. However, based on univariate analyses, no significant differences in plasma LRP1 levels were observed in relation to MIAC, IAI, and microbial-associated IAI (Table 1). Interactions between plasma FCGR3A, haptoglobin, and LRP1 levels were not found for the presence of MIAC, IAI, and microbial-associated IAI (Table S8).

**Table 1:** Various plasma proteins of the study population according to the presence or absence of microbial invasion of the amniotic cavity (MIAC), intra-amniotic inflammation (IAI), and microbial-associated IAI in women with preterm premature rupture of membranes.

	MIAC		*P-*value	IAI		*P*-value	Microbial-associated IAI		*P*-value
	Positive (*n* = 63)	Negative (*n* = 119)		Positive (*n* = 78)	Negative (*n* = 104)		Positive (*n* = 52)	Negative (*n* = 130)	
Plasma FCGR3A (ng/mL)	85.44 ± 83.73	52.62 ± 72.27	**0.001**	72.86 ± 79.99	57.33 ± 75.81	**0.022**	83.68 ± 82.55	56.11 ± 74.69	**0.004**
Plasma haptoglobin (mg/mL)	0.67 ± 0.43	0.53 ± 0.37	**0.046**	0.69 ± 0.47	0.49 ± 0.29	**0.004**	0.70 ± 0.44	0.53 ± 0.37	**0.014**
Plasma LRP1 (ng/mL)	2.80 ± 5.04	2.84 ± 4.54	0.475	2.84 ± 5.23	2.82 ± 4.31	0.180	2.66 ± 4.94	2.90 ± 4.63	0.270

*FCGR3A* low affinity immunoglobulin gamma Fc region receptor III-A, *LRP1* prolow-density lipoprotein receptor-related protein 1. Data are given as mean ± standard deviation.

Significant values are in [bold].

**Table 2:** Demographic and clinical characteristics of the study population according to the presence or absence of microbial invasion of the amniotic cavity (MIAC), intra-amniotic inflammation (IAI), and microbial-associated IAI in women with preterm premature rupture of membranes.

	MIAC		*P*-value	IAI		*P*-value	Microbial-associated IAI		*P*-value
	Positive (*n* = 63)	Negative (*n* = 119)		Positive (*n* = 78)	Negative (*n* = 104)		Positive (*n* = 52)	Negative (*n* = 130)	
Maternal age (years)	32.1 ± 3.7	31.4 ± 3.9	0.195	32.1 ± 3.7	31.4 ± 4.0	0.287	31.9 ± 3.7	31.6 ± 3.9	0.574
Nulliparity	39.7% (25/63)	50.4% (60/119)	0.167	35.9% (28/78)	54.8% (57/104)	**0.011**	36.5% (19/52)	50.8% (66/130)	0.082
Gestational age at sampling (weeks)	29.4 ± 3.0	30.3 ± 2.9	**0.023**	28.9 ± 3.1	30.8 ± 2.6	** < 0.001**	29.3 ± 2.8	30.3 ± 3.0	**0.008**
Gestational age at delivery (weeks)	30.7 ± 2.5	32.9 ± 2.6	** < 0.001**	30.6 ± 2.6	33.2 ± 2.3	** < 0.001**	30.4 ± 2.4	32.8 ± 2.6	** < 0.001**
Serum CRP (mg/dL)	1.2 ± 1.5	0.7 ± 1.2	**0.024**	1.5 ± 1.8	0.5 ± 0.6	** < 0.001**	1.4 ± 1.6	0.7 ± 1.2	**0.007**
Use of tocolytic agents	63.5% (40/63)	58.0% (69/119)	0.471	66.7% (52/78)	54.8% (57/104)	0.106	69.2% (36/52)	56.2% (73/130)	0.104
Use of antibiotics	98.4% (62/63)	93.3% (111/119)	0.166	94.9% (74/78)	95.2% (99/104)	1.000	98.1% (51/52)	93.8% (122/130)	0.450
Use of antenatal corticosteroids	93.7% (59/63)	88.2% (105/119)	0.304	92.3% (72/78)	88.5% (92/104)	0.390	92.3% (48/52)	89.2% (116/130)	0.784
Clinical chorioamnionitis	11.1% (7/63)	8.4% (10/119)	0.550	9.0% (7/78)	9.6% (10/104)	0.883	11.5% (6/52)	8.5% (11/130)	0.519
Histological chorioamnionitis^a^	74.1% (43/58)	41.3% (43/104)	** < 0.001**	73.2% (52/71)	37.4% (34/91)	** < 0.001**	77.1% (37/48)	43.0% (49/114)	** < 0.001**

CRP, C-reactive protein. Data are given as mean ± standard deviation or % (n/N).

^a^Data for the histologic evaluation of the placenta were only available in 162 of the 182 women because in 17 cases, delivery took place at another institution and in 3 cases, histologic evaluation of the placenta was not performed because of our institutional policy that only the placentas in cases of preterm delivery are to be sent for histopathologic examination or because of missing data for the histological chorioamnionitis.

Significant values are in [bold].

**Table 3:** Relationship of various plasma proteins with the presence of microbial invasion of the amniotic cavity (MIAC), intra-amniotic inflammation (IAI), and microbial-associated IAI, analyzed using multiple logistic regression.

Variables	MIAC^a^		IAI^b^		Microbial-associated IAI^b^	
	OR (95% CI)	*P-*value	OR (95% CI)	*P-*value	OR (95% CI)	*P-*value
Plasma FCGR3 (ng/mL)	1.006 (1.002–1.010)	**0.007**	1.003 (0.999–1.007)	0.160	1.005 (1.000–1.009)	**0.028**
Plasma haptoglobin (mg/mL)	2.354 (1.078–5.143)	**0.032**	5.189 (2.068–13.020)	** < 0.001**	2.833 (1.253–6.407)	**0.012**

*OR* odds ratio, *CI* confidence interval, *FCGR3A* low affinity immunoglobulin gamma Fc region receptor III-A.

^a^Adjustment for gestational age at sampling.

^b^Adjustment for gestational age at sampling and parity.

Significant values are in [bold].

**Figure 3:**
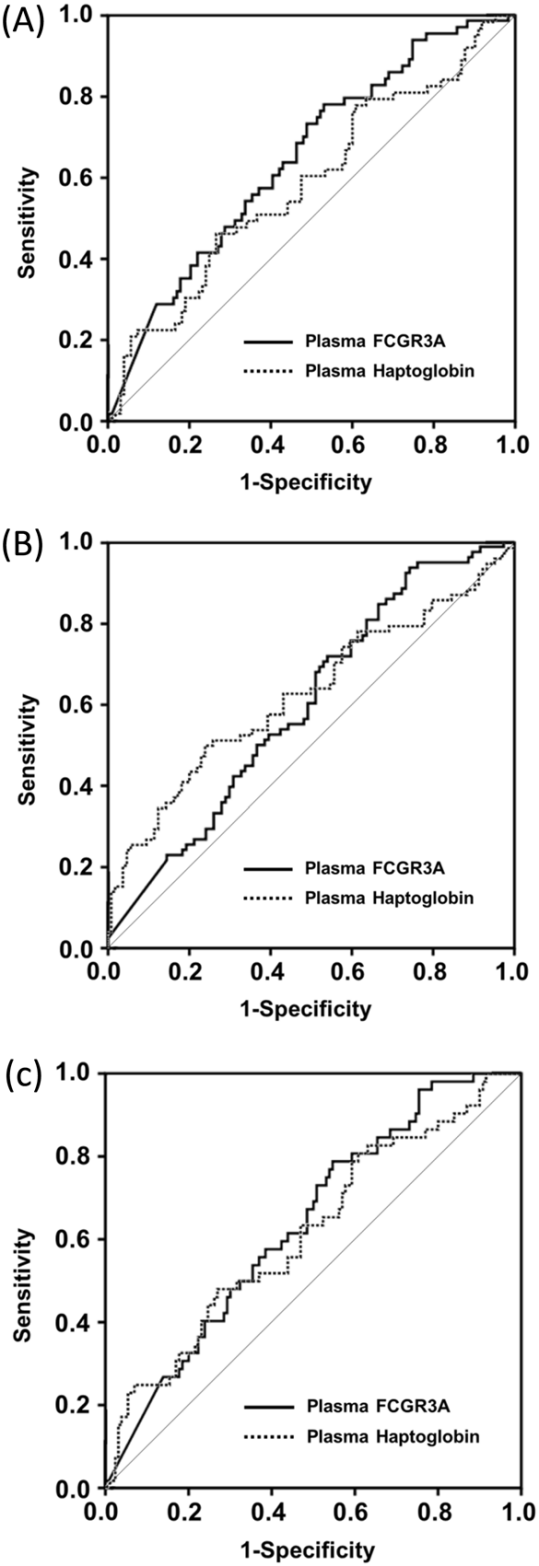
ROC curves of plasma FCGR3A and haptoglobin for preicting (**A**) MIAC, (**B**) IAI, and (**C**) microbial-associated IAI. *ROC* receiver-operating characteristic, *FCGR3A* low affinity immunoglobulin gamma Fc region receptor III-A, *MIAC* microbial invasion of the amniotic cavity, *IAI* intra-amniotic inflammation.

The AUC values of plasma FCGR3A and haptoglobin for predicting MIAC were 0.65 and 0.59, and for IAI were of 0.60 and 0.63, respectively (Table 4 and Fig. 3A,B). Similarly, for the prediction of microbial-associated IAI, the AUC values of plasma FCGR3A and haptoglobin were of 0.64 and 0.62, respectively (Table 4 and Fig. 3C). Differences in the AUC values between plasma FCGR3A and haptoglobin were not statistically significant for predicting any of the three outcome measures (*P* = 0.370–0.758). Moreover, the AUC values of plasma FCGR3A and haptoglobin were similar to those of serum CRP with respect to each of the corresponding outcome measures (*P* = 0.299–0.879).

**Table 4:** Diagnostic indices of various plasma biomarkers to predict microbial invasion of the amniotic cavity, intra-amniotic inflammation, and microbial-associated intra-amniotic inflammation.

Variables	Area (± SE) under the ROC curve	95% CI	Cut-off value^a^	Sensitivity^b^ (95% CI)	Specificity^b^ (95% CI)	PPV	NPV
Microbial invasion of the amniotic cavity							
Plasma FCGR3 (ng/mL)	0.65 ± 0.04	0.56–0.73	7.16	77.8 (65.5–87.3)	47.1 (37.9–56.4)	43.8	80.0
Plasma haptoglobin (mg/mL)	0.59 ± 0.05	0.50–0.68	0.66	46.0 (33.4–59.1)	74.0 (65.1–81.6)	48.3	72.1
Serum CRP (mg/dL)	0.60 ± 0.05	0.51–0.69	0.58	49.2 (36.4–62.1)	71.8 (62.7–79.7)	48.4	72.4
Combined model A^c^	0.68 ± 0.04	0.59–0.76	0.27	77.8 (65.5–87.3)	53.0 (43.6–62.3)	47.1	81.6
Intra-amniotic inflammation							
Plasma FCGR3 (ng/mL)	0.60 ± 0.04	0.52–0.68	8.04	69.2 (57.8–79.2)	48.1 (38.2–58.1)	50.0	67.6
Plasma haptoglobin (mg/mL)	0.63 ± 0.04	0.54–0.71	0.64	50.0 (38.5–61.5)	76.0 (66.6–83.8)	60.9	67.0
Serum CRP (mg/dL)	0.67 ± 0.04	0.59–0.75	0.58	50.0 (38.5–61.5)	75.5 (65.9–83.5)	60.9	66.4
Microbial-associated intra-amniotic inflammation							
Plasma FCGR3 (ng/mL)	0.64 ± 0.04	0.55–0.72	7.16	78.9 (65.3–88.9)	45.4 (36.6–54.4)	36.6	84.3
Plasma haptoglobin (mg/mL)	0.62 ± 0.05	0.53–0.71	0.66	48.1 (34.0–62.4)	73.1 (64.6–80.5)	41.7	77.9
Serum CRP (mg/dL)	0.63 ± 0.05	0.53–0.73	0.58	53.8 (39.5–67.8)	71.9 (63.3–79.5)	43.8	79.3
Combined model B^d^	0.69 ± 0.04	0.61–0.78	0.22	84.6 (71.9–93.1)	53.1 (44.1–62.0)	42.3	89.5

*SE* standard error, *ROC* receiver-operating characteristic, *CI* confidence interval, *PPV* positive predictive value, *NPV* negative predictive value, *FCGR3A* low affinity immunoglobulin gamma Fc region receptor III-A, *CRP* C-reactive protein.

^a^Cut-off values corresponding to the highest sum of sensitivity and specificity.

^b^Values are given as % (95% CI).

^c^Combined model A consists of plasma FCGR3 and serum CRP levels.

^d^Combined model B consists of plasma FCGR3 and serum CRP levels.

Repetition of the univariate and multivariate analyses after excluding data that had been included in the discovery study (*n* = 164), the results for each outcome measure were confirmed, being the same as those observed in the total cohort (*n* = 182) (Tables S5, S6, and S7). No correlations were found among the measured plasma proteins (FCGR3A, haptoglobin, and LRP1) (all variables, r =  − 0.078 to 0.051, *P* > 0.2).

In the multi-marker panel for the diagnosis of MIAC, plasma FCGR3 and serum CRP levels were identified as the best combination (Table S9), with an AUC value of 0.68 (95% confidence interval [CI]: 0.59–0.76; *P* = 0.380 by Hosmer–Lemeshow test), which was not significantly higher than those for plasma FCGR3 and serum CRP (*P* = 0.373 and 0.107, respectively) (Table 4). Similarly, for predicting microbial-associated IAI, plasma FCGR3 levels along with serum CRP levels were identified as the best combination (Table S9), with an AUC value of 0.69 (95% CI: 0.61–0.78; *P* = 0.184 by Hosmer–Lemeshow test). The AUC value for this two-biomarker panel was similar to those of plasma FCGR3 and serum CRP (*P* = 0.141 and 0.156, respectively) (Table 4). However, in the IAI predictive model, plasma FCGR3A, plasma haptoglobin, and serum CRP levels were set in the logistic regression model as predictors, but only serum CRP level was selected for the best multi-marker panel; thus, a predictive model for IAI could not be generated.

## Discussion

In the current study of women with PPROM, (i) 17 significant plasma DEPs related to MIAC/IAI and their potential biological pathways were identified using label-free shotgun proteomics approaches; and (ii) these proteomic findings were further validated and confirmed by ELISA. In particular, FCGR3A and haptoglobin were found to be significantly elevated in the plasma of women with MIAC, IAI, and both MIAC/IAI compared with those without these conditions. To the best of our knowledge, this is the first report demonstrating a comprehensive profile of the plasma proteome related to MIAC/IAI in patients with PPROM using a high-throughput shotgun label-free quantitation approach. The present study provides new insights for a better understanding of the biochemical mechanisms and molecular signals occurring in maternal circulation that are associated with inflammatory/infectious processes in the amniotic cavity.

In the last two decades, several investigations employed proteomic approaches to explore biomarkers for MIAC/IAI complicated by PPROM or PTL in AF and cervicovaginal fluid samples^18–20,38–40^. They reported several new proteins, including α1-acid glycoprotein, IGFBP-1, calgranulins, lipocalin-2, myeloperoxidase, neutrophil protein 1–3, as being associated with MIAC/IAI. However, to date, no study employed proteomic techniques in maternal blood to screen for potential biomarkers of MIAC/IAI. In our plasma proteomics and immunoassays, for the first time, we identified plasma FCGR3A and haptoglobin as novel biomarkers that may be used to differentiate MIAC/IAI from non-MIAC/IAI patients complicated by PPROM.

FCGR3A, which is also known as CD16A, is a transmembrane glycoprotein receptor that is member of the Fc gamma receptor family. It is one of the major receptors for IgG and is expressed on natural killer cells, monocytes/macrophages, trophoblasts, and dendritic cells^41–43^. FCGR3A is involved in antibody-dependent cell-mediated cytotoxicity, cytokine production, phagocytosis, and removal of antigen–antibody complexes^44^. Mutations and aberrant expression of FCGR3A have been reported to contribute to increased susceptibility to several inflammatory and immune diseases, including systemic lupus erythematosus, recurrent infectious diseases, and childhood chronic immune thrombocytopenic purpura^45–47^. Indeed, Presicce et al*.* showed that intraamniotic injection of lipopolysaccharide increases FCGR3 expression in chorio-decidua neutrophils in rhesus macaques^48^. Negishi et al*.* also found that the accumulation of FCGR3^+^ natural killer cells is increased in the decidua basalis of women who have experienced late preterm birth with acute chorioamnionitis^49^. The results of the above studies, along with the main biological characteristics of FCGR3A, support and in general agree with the plasma FCGR3 data in the present study, considering that significant associations have been reported between MIAC/IAI and acute HCA/SPTD risk in the PPROM context^26,27,50^.

Haptoglobin is an acute-phase protein that is produced mainly by the liver in response to proinflammatory stimuli, such as IL-1, IL-6, and tumor necrosis factor (TNF)-α^51^. Haptoglobin binds to free hemoglobin and matrix metalloproteinase-9 in circulation^52^, acts as an antioxidant, exhibits proangiogenic and antibacterial activities, and plays an important role in innate immunity^51^. In line with its known biological properties in inflammation, haptoglobin has been reported to be elevated in the plasma of patients with various inflammatory conditions, including inflammatory, infectious, and malignant diseases, and diabetes^51^. Particularly in the perinatal field, previous studies have shown that haptoglobin expression is upregulated in the cord blood or placenta of newborns with early onset neonatal sepsis and antenatal exposure to inflammation/infection (acute HCA, MIAC, and IAI) compared with those without these conditions^53,54^. Furthermore, a previous study by Oggé et al. showed that haptoglobin expression is upregulated in the AF of women with chronic HCA compared with controls^55^. However, haptoglobin expression has not yet been evaluated in maternal blood from women with PPROM with respect to MIAC/IAI. In the present study, we demonstrated for the first time that higher plasma haptoglobin levels are independently associated with the occurrence of MIAC/IAI in pregnancies complicated by PPROM.

Noteworthily, despite the fact that plasma FCGR3A and haptoglobin can discriminate MIAC/IAI from non-MIAC/IAI among a cohort of women with PPROM with similar diagnostic accuracy to serum CRP (a prototype marker of inflammation tested in the blood), their diagnostic performance is poor to fair (AUC: 0.59–0.65; Table 4). These observations are similar to those reported for other maternal blood biomarkers and may be attributed to the common inherent characteristics of non-specific inflammation biomarkers^12,26,56^. Consequently, the clinical utility of the aforementioned plasma biomarkers alone may be limited in the PPROM setting; thus, whether these newly identified plasma-based biomarkers could contribute to identifying PPROM-associated MIAC/IAI when used in combination with other currently available non-invasive tests (*e.g.,* cervical length and inflammatory cytokines in the cervicovaginal fluid) needs to be addressed in future studies^5,12,57,58^. In addition, considering the significant association between the inflammatory response in circulation and SPTD^59–61^, it is highly likely that these two biomarkers may be useful for identifying patients at high risk for infection/inflammation-associated preterm delivery, which deserves to be the focus of future studies.

In the present study, IPA revealed the most significant canonical pathways associated with the 17 DEPs identified in maternal plasma potentially involved in MIAC/IAI. Overall, the five top signaling pathways identified are parallel to those associated with SPTD development, as reported in previous proteomics studies on patients at risk for preterm delivery^62–65^. APR is a complex systemic inflammatory reaction triggered by various factors, including local infection/inflammation, trauma, and tissue damage^66^. APR is mediated by proinflammatory cytokines (notably IL-1, IL-6, and TNF-α), leading to the release of acute-phase proteins from hepatocytes into the plasma^67,68^. Previous studies have shown that the levels of proinflammatory cytokines are significantly elevated in the AF and maternal plasma (or serum) from women with MIAC/IAI complicated by PPROM, and that simultaneously increased levels of various acute-phase proteins occur in the blood during this condition^12,23,56^. Thus, it is natural that the APR signaling pathway plays an important role in the link between local infection/inflammation in the amniotic cavity and systemic inflammatory responses. In growth hormone signaling, growth hormones play an important role in the regulation of growth and metabolism (glucose and lipid) during development, which is mediated through insulin-like growth factor (IGF) 1 and 2^69^, as well as the immune system^70^. Furthermore, reduced growth hormone levels are associated with increased plasma secretion of proinflammatory cytokines (such as IL-6 and TNF-α) and increased proinflammatory function of monocytes/macrophages^69,71^. Iron homeostasis acts as an essential regulator of adaptive and innate immunity, and plays a crucial role in inflammatory processes and immune responses, especially during infection^72^. In particular, immune cells (including B cells, T cells, and macrophages) require sufficient amounts of iron to sustain their development and effector functions; thus, iron deficiency robustly impairs T and B cell activation, and the production and release of cytokines^72^. The PPAR forms a heterodimeric DNA-binding complex with RXR, which is called “PPAR/RXR activation,” that plays a critical role in energy balance, including lipid metabolism and glucose homeostasis, and is also involved in the inflammatory and vascular responses^73^. HIF-1 signaling pathway is activated mainly by hypoxia and is modulated by cytokines (*e.g*., ILs) and growth factors (*e.g*., IGFs), and has been implicated in the regulation of inflammatory response, tumor progression, and metastasis^74,75^.

Our proteomic study was performed using semi-pooled samples (three pools containing three, three, or three samples in each pool). Noteworthy, sample pooling strategy for proteomic analysis have some disadvantages: (i) may not represent the biological average of individual samples, and (ii) reduces the statistical value of the identified biomarkers (false identification of biomarkers and their missed detection than when using individual samples)^76^. However, compared with the individual sample strategy, it provides the following advantages: (i) reduced biological variation, especially for individual proteomes not associated with MIAC/IAI; (ii) requires a small amount of samples for analysis; and (iii) reduced experimental time and cost^76,77^. Thus, the semi-pooling strategy herein used may compensate for the drawbacks inherent to pooling all 9 samples from each group together, as well as provide three independent biological replicates for each group^78,79^.

The current study had several limitations. First, conventional microbial culture-based techniques were used only for microorganism detection in AF specimens, which may have led to false-negative MIAC results. A combination of culture and non-culture methods, including *16S* rDNA polymerase chain reaction, provide an opportunity to precisely identify and characterize the microbes in AF^80^. Second, stored (at − 70 °C) plasma samples were used, which may have affected the results of proteomic and immunoassay analyses owing to protein degradation caused by long-term storage^81–83^. Third, the retrospective nature of our study had inherent drawbacks, including selection bias, and the validation study of candidate biomarkers and clinical validation of cutoff values were not conducted in a completely independent dataset. All these factors may limit the generalizability of the present findings, which may warrant further validation in other cohorts. Fourth, the label-free quantification strategy based on peak intensity herein used may result on considerably lower quantification accuracy and precision than label-based quantitation methods^84^. Nevertheless, the label-free approach has the key advantage of high proteome coverage and dynamic range, as well as no restrictions on sample type^84^. The major strengths of the present study are as follows: (i) a relatively large sample and cohort size; (ii) a relatively high rate for which proteins identified as DEPs in this proteomic study were reproduced in the results of the ELISA (2/3, 66.6%), which suggests that the shotgun proteomics experiments were properly conducted; and (iii) identification of MIAC/IAI-specific protein biomarkers in a less-invasive, but still complex biological sample (*i.e*., plasma), which is often challenging owing to the wide dynamic range of proteins, and high levels of salts and other interfering compounds in such samples^85^.

The discrepancy concerning the results of LRP1 between the shotgun proteomics and ELISA data can be generally found in other proteomics-based biomarker discovery and verification studies^19,62,64,86–88^, but this disagreement may be attributed to (i) sample types evaluated (pooled *vs*. individual samples), (ii) somewhat loose threshold used in the present study as selection criteria for DEPs (fold change of LFQ intensities > 1.3 or < 0.77), and (iii) inherent disadvantages of the ELISA method, including cross-reactivity of the antibodies with non-target antigens, high-dose hook effect, and that the target proteins might be denatured during ELISA processing and thus their epitopes cannot be detected by the secondary antibodies^89,90^.

## Conclusions

In summary, using proteomic approaches, 17 DEPs were identified as novel potential candidate biomarkers for MIAC/IAI in plasma samples collected from women with PPROM. In particular, plasma FCGR3A and haptoglobin were confirmed by ELISA as potential independent biomarkers for less-invasive identification of MIAC/IAI. Nevertheless, these biomarkers alone exhibited poor-to-fair diagnostic performance for PPROM-associated MIAC/IAI. The possible mechanistic roles of FCGR3A and haptoglobin in maternal circulation as pathophysiological links with inflammation/infection in the amniotic cavity warrant further investigation. Further studies are warranted to examine whether the alteration of these two proteins in other biological samples, such as cervicovaginal fluid, AF, saliva, or urine, could be used to identify MIAC/IAI risk.

## Supplementary Information

Supplementary Information 1.

Supplementary Information 2.

Supplementary Information 3.

## Data Availability

All relevant data are within the paper, and the authors can make available materials, data and associated protocols if requested.
