# Supplementary material for: Proteomic analysis of plasma to identify novel biomarkers for intra-amniotic infection and/or inflammation in preterm premature rupture of membranes
Source: Sci Rep. 2023 Apr 6;13:5658. doi: 10.1038/s41598-023-32884-y (PMC10079851; doi:10.1038/s41598-023-32884-y)
Supplement: Supplementary file 2 — Supplementary Information 2. [file 41598_2023_32884_MOESM2_ESM.docx]

**Title page**

**Proteomic analysis of plasma to identify novel biomarkers for intra-amniotic infection and/or inflammation in preterm premature rupture of membranes**

Ji Hyun Back^1,2,†^, So Yeon Kim^3,†^, Man Bock Gu^1^, Hyeon Ji Kim^4^, Kyong-No Lee^4^, Ji Eun Lee^2,*^, and Kyo Hoon Park^4,*^

^1^Department of Biotechnology, College of Life Sciences and Biotechnology, Korea University, Seoul 02841, Korea

^2^Chemical & Biological integrative Research Center, Biomedical Research Division, Korea Institute of Science and Technology, Seoul 02792, Korea

^3^Department of Obstetrics and Gynecology, University of Ulsan College of Medicine, Asan Medical Center, Seoul, Korea.

^4^ Department of Obstetrics and Gynecology, Seoul National University College of Medicine, Seoul National University Bundang Hospital, Seongnam, Korea

^†^ These two authors contributed equally to this work and should therefore be regarded as equivalent authors.

**^*^** Corresponding author

Address correspondence to:

Kyo Hoon Park, MD, PhD**^*^**

Department of Obstetrics and Gynecology

Seoul National University Bundang Hospital

82, Gumi-ro 173 Beon-gil, Bundang-gu, Seongnam, 463-707, Korea

Tel: 82-31-787-7252; Fax: 82-31-787-4054; E-mail: [pkh0419@snubh.org](mailto:pkh0419@snubh.org)

Ji Eun Lee, PhD**^*^**

Chemical & Biological integrative Research Center, Biomedical Research Division, Korea Institute of Science and Technology, Seoul 02792, Korea

Tel: 82-2-958-6422; Fax: 82-2-958-5308; E-mail: [jelee9137@](mailto:jelee9137@)kist.re.kr

## - Supplementary Materials -

**Analysis of IL-6 in the AF and diagnosis of clinical chorioamnionitis**

The range of the IL-6 standard curve was 7.8–600 pg/mL. The assay was carried out by strictly following the instructions provided by the manufacturer and samples were measured in duplicate. The calculated intra- and inter-assay coefficients of variation (CV) were <10%. Clinical chorioamnionitis was diagnosed following the criteria proposed by Gibbs et al.^2^; fever (≥37.8°C) and the presence of two or more of the associated clinical findings (uterine tenderness, malodorous vaginal discharge, maternal leukocytosis, maternal tachycardia, and fetal tachycardia).

**Pooling of plasma samples**

**Aliquots of the frozen plasma were thawed in water bath (Branson 2510 Ultrasonic Cleaner, Branson Ultrasonics Corporation, Brookfield, CT, USA) at room temperature for 1 min and were stored on ice.** After protease inhibitor cocktail (Roche Diagnostics, Mannheim, Germany) was added to 18 plasma samples consisting of No-MIAC/Non-IAI controls (n = 9) and MIAC/IAI cases (n = 9), protein concentrations of each plasma samples were determined using a bicinchoninic acid (BCA) protein assay kit (Thermo Fisher Scientific, Bremen, Germany). Then, the three individual plasma samples were pooled with equal amounts (3,333.33 μg) in each group, resulting in three sets of pooled samples for No-MIAC/Non-IAI (control) and MIAC/IAI (case) groups. A total of 10,000 μg of the pooled plasma samples (three sets of pooled samples per group) were subjected to immunoaffinity depletion.

**Immunoaffinity depletion of high-abundance proteins**

The top 14 high-abundance proteins (albumin, alpha-1 acid glycoprotein, alpha-1 antitrypsin, alpha-2 macroglobulin, apolipoprotein A-I, apolipoprotein A-II, complement C3, fibrinogen, haptoglobin, immunoglobulin A, immunoglobulin G, immunoglobulin M, serotransferrin, and transthyretin) were removed from each pooled plasma sample (10,000 μg) using a human multiple affinity removal system (MARS)-14 column (4.6 × 50 mm, Agilent Technologies, Santa Clara, CA) according to the manufacturer’s instructions. The depleted samples were concentrated using Amicon Ultracel-3 centrifugal filter devices (3 kDa cutoff, Millipore, Billerica, MA) and the protein concentrations of the depleted plasma samples were determined using the BCA protein assay kit.

**In-solution tryptic digestion**

Four hundred and fifty micrograms of total protein from each depleted plasma sample was resuspended in 100 μL of 8 M urea buffer and reduced with 5 mM tris (2-carboxyethyl) phosphine hydrochloride for 1 h at 37 °C to reduce cysteine residues followed by addition of 10 mM iodoacetamide at room temperature for 1 h in the dark to alkylate the cysteine residues in the proteins. The samples were then diluted with 25 mM ammonium bicarbonate to decrease the urea concentration to less than 1 M and digested with lysyl endopeptidaseR (Lys-C, Fujifilm Wako Pure Chemical Corporation, Osaka, Japan) in an enzyme/substrate ratio of 1 mAU Lys-C per 50 μg of total protein at 25 °C for 2 h.^1^ Then, trypsin (Promega, Madison, WI, USA) was added to the samples in an enzyme/substrate ratio of 1:50 (wt/wt) and incubated at 37 °C overnight. The digested peptide samples were acidified with 10% trifluoroacetic acid and desalted using a Sep-Pak tC18 cartridge (Waters Corporation, Milford, MA, USA). The desalted peptide samples were dried in a miVAC vacuum concentrator (Genevac Ltd, Ipswich, UK).

**Basic pH reversed-phase liquid chromatography**

The dried peptide samples were resuspended in 10 mM ammonium formate and the peptide concentrations were determined using a quantitative colorimetric peptide assay kit (Thermo Fisher Scientific, Waltham, MA, USA). Then, the peptides were fractionated by high pH reversed-phase liquid chromatography using an Agilent 1290 Infinity liquid chromatography (LC) system (Agilent Technology, Santa Clara, CA). Two hundred and fifteen micrograms of the peptides from each sample were loaded onto an X-Bridge peptide BEH C18 column (4.6 mm i.d. × 250 mm length; pore size 130 Å; particle size 3.5μM, Waters Corporation, Milford, MA, USA) and separated at a flow rate of 0.5 mL/min with following gradient conditions: 0 min 100% buffer A (10 Mm ammonium formate, pH 10) and 0% buffer B [10 mM ammonium formate (pH 10) in 90% acetonitrile], 0-10 min 0-5% B, 10-48.5 min 5-40% B, 48.5-62.5 min 40-70% B, 62.5-72.5 min 70% B, 72.5-82.5 min 70-5% B, and 82.5-92.5 min 5% B. Fractionation was pursued by collecting 96 wells (1 well/0.8 min, Restek corporation, Bellefonte, PA, USA) during the chromatographic run (from 10 min to 82.5 min). The resultant 96 fractions were pooled to 24 concatenated fractions by the following rule. A set of an arithmetic sequence with a common difference of 24 was pooled into one concatenated fraction, for example, number 1, 25, 49, and 63 fractions were pooled into the first concatenated faction. The peptide fractions were dried and subsequently resuspended in 35.83 μL of 0.4% acetic acid.

**Liquid chromatography and tandem mass spectrometry (LC-MS/MS) analysis**

The fractionated peptide samples were analyzed in triplicate on a reversed-phase Magic C18AQ column (15 cm × 75 µm) using an Eksigent MDLC system (Eksigent Technologies Dublin, CA, USA). The operating flow rate was 350 nL/min with the following gradient conditions: 0 min 100% buffer A (100% water with 0.1% formic acid) and 0% buffer B (100% acetonitrile with 0.1% formic acid), 0-5 min 0-8% B, 5-85min 8-30% B, 85-90 min 30-70% B, 90-100 min 70% B, 100-110 min 70-2% B, and 100-120 min 2% B. The nano HPLC system was coupled to an LTQ XL-Orbitrap mass spectrometer (Thermo Fisher Scientific, Waltham, MA, USA). Survey full-scan mass spectrometry (MS) spectra (300–1800 *m/z*) were acquired at a resolution of 60,000, allowing preview mode for precursor selection and charge-state determination. Tandem mass (MS/MS) spectra for the ten most intense ions from the preview survey scan were acquired concurrently in the ion trap with the following options: isolation width, 2 m/z; normalized collision energy, 35%; dynamic exclusion duration, 360 s. Precursors with +1 charge and unassigned charge states were discarded during data-dependent acquisition.

**Protein identification and quantification**

Protein identification was pursued using MaxQuant (version 1.6.7.0) software; the search criteria were set to an initial search with a precursor mass tolerance of 20 ppm for mass recalibration and a main search with precursor mass and fragment mass tolerance of 15 ppm and 0.5 Da, respectively. The search included fixed modification of carbamidomethylation of cysteine (+57.021 Da) and variable modification of methionine oxidation (+15.995 Da). The ‘match between runs’ feature was activated. The relative abundances of proteins were calculated based on peak intensities provided from the LFQ feature of MaxQuant (MaxLFQ). For statistical analysis of the dataset obtained from the label-free quantification using Perseus software (1.6.14.0), the LFQ intensity values were log-transformed. Missing values were replaced using values computed from the normal distribution with a width of 0.3 and a downshift of 1.8. Statistically significant proteins between control and case groups were found by Student’s *t*-test comparison of the log_2_ (LFQ intensity) values obtained from the three replicates of each type of plasma samples. A *P*-value <0.05 was considered statistically significant. For hierarchical clustering of proteins showing statistically significant changes (>1.3-fold, *P*-value <0.05) between No-MIAC/Non-IAI (control) and MIAC/IAI (case) groups, log_2_ (LFQ intensity) values were first normalized using z-score and then clustering of both columns and rows was pursued based on Euclidean distance using the average linkage method using Perseus (1.6.14.0).

**Analysis of various proteins in the amniotic fluid**

The ranges of the FCGR3A, haptoglobin, and LRP1 standard curves were 31.3 – 2000 pg/mL, 31.3 – 2000 pg/mL, and 0.2 – 10 ng/mL, respectively. Prior to measurement of these three proteins, the maternal plasma samples were diluted at 1:2 for LRP1, 1:10 for FCGR3A, and 1:1,000,000 for haptoglobin. The intra- and inter-assay coefficients of variation were 9.9% and 11.2% for FCGR3A, 5.0% and 11.4% for haptoglobin, and 2.4% and 3.9% for LRP1, respectively.

**Reference**

1. Reproducible workflow for multiplexed deep-scale proteome and phosphoproteome analysis of tumor tissues by liquid chromatography–mass spectrometry. Nat Protoc. 2018 July ; 13(7): 1632–1661. doi:10.1038/s41596-018-0006-9).
