# Supplementary material for: Proteomic analysis of plasma to identify novel biomarkers for intra-amniotic infection and/or inflammation in preterm premature rupture of membranes
Source: Sci Rep. 2023 Apr 6;13:5658. doi: 10.1038/s41598-023-32884-y (PMC10079851; doi:10.1038/s41598-023-32884-y)
Supplement: Supplementary file 3 — Supplementary Information 3. [file 41598_2023_32884_MOESM3_ESM.pdf]

Title page

Proteomic analysis of plasma to identify novel biomarkers for intra-amniotic infection and/or inflammation in preterm premature rupture of membranes

Ji Hyun Back[1,2,†], So Yeon Kim[3,†], Man Bock Gu[1], Hyeon Ji Kim[4], Kyong-No Lee[4], Ji Eun Lee[2,*], and Kyo Hoon Park[4,*]

[1]Department of Biotechnology, College of Life Sciences and Biotechnology, Korea University, Seoul 02841, Korea

[2]Chemical & Biological integrative Research Center, Biomedical Research Division, Korea Institute of Science and Technology, Seoul 02792, Korea

[3]Department of Obstetrics and Gynecology, University of Ulsan College of Medicine, Asan Medical Center, Seoul, Korea.

[4]Department of Obstetrics and Gynecology, Seoul National University College of Medicine, Seoul National University Bundang Hospital, Seongnam, Korea

[†] These two authors contributed equally to this work and should therefore be regarded as equivalent authors.

[*] Corresponding author

Address correspondence to:

Kyo Hoon Park, MD, PhD[*]

Department of Obstetrics and Gynecology

Seoul National University Bundang Hospital

82, Gumi-ro 173 Beon-gil, Bundang-gu, Seongnam, 463-707, Korea

Tel: 82-31-787-7252; Fax: 82-31-787-4054; E-mail: pkh0419@snubh.org

Ji Eun Lee, PhD[*]

Chemical & Biological integrative Research Center, Biomedical Research Division, Korea Institute of Science and Technology, Seoul 02792, Korea

Tel: 82-2-958-6422; Fax: 82-2-958-5308; E-mail: jelee9137@kist.re.kr

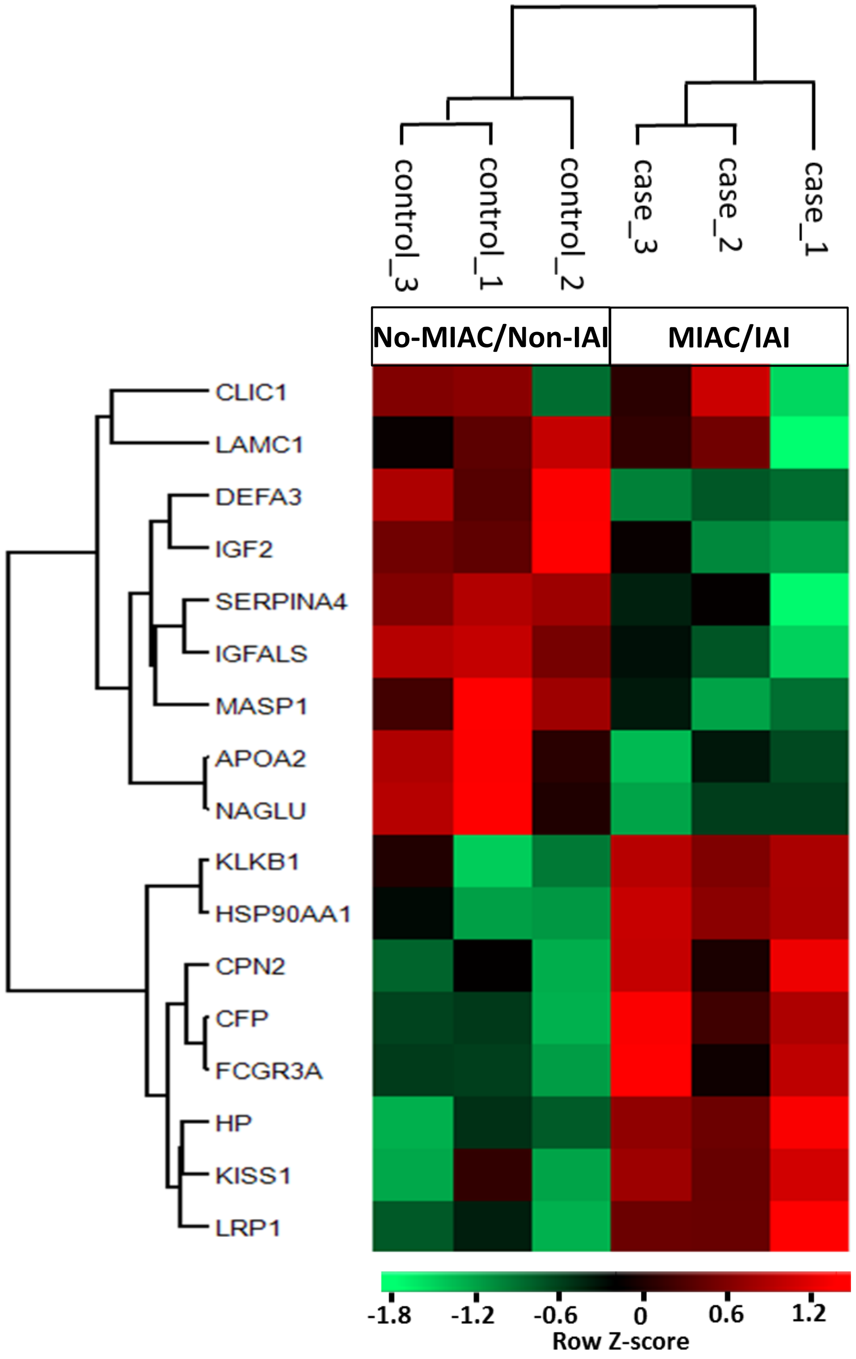

Figure S1. Heatmap from the shotgun proteomics analysis. Heatmap and hierarchical clustering analysis of 17 statistically significant DEPs between MIAC/IAI case and No-MIAC/Non-IAI control groups. Red and green indicate upregulated and downregulated levels, respectively. DEP, differentially expressed proteins.
